# Tri-Trophic Effects of Seasonally Variable Induced Plant Defenses Vary across the Development of a Shelter Building Moth Larva and Its Parasitoid

**DOI:** 10.1371/journal.pone.0120769

**Published:** 2015-03-17

**Authors:** Noah H. Rose, Rayko Halitschke, Douglass H. Morse

**Affiliations:** 1 Department of Ecology and Evolutionary Biology, Box G-W, Brown University, Providence, Rhode Island, 02912, United States of America; 2 Department of Ecology and Evolutionary Biology, Cornell University, E4435 Corson Hall, Ithaca, New York, 14853, United States of America; Swedish University of Agricultural Sciences, SWEDEN

## Abstract

Plant chemical defenses can negatively affect insect herbivore fitness, but they can also decrease herbivore palatability to predators or decrease parasitoid fitness, potentially changing selective pressures on both plant investment in production of chemical defenses and host feeding behavior. Larvae of the fern moth *Herpetogramma theseusalis* live in and feed upon leaf shelters of their own construction, and their most abundant parasitoid *Alabagrus texanus* oviposits in early instar larvae, where parasitoid larvae lay dormant for most of host development before rapidly developing and emerging just prior to host pupation. As such, both might be expected to live in a relatively constant chemical environment. Instead, we find that a correlated set of phenolic compounds shows strong seasonal variation both within shelters and in undamaged fern tissue, and the relative level of these compounds in these two different fern tissue types switches across the summer. Using experimental feeding treatments, in which we exposed fern moth larvae to different chemical trajectories across their development, we show that exposure to this set of phenolic compounds reduces the survival of larvae in early development. However, exposure to this set of compounds just before the beginning of explosive parasitoid growth increased parasitoid survival. Exposure during the period of rapid parasitoid growth and feeding decreased parasitoid survival. These results highlight the spatial and temporal complexity of leaf shelter chemistry, and demonstrate the developmental contingency of associated effects on both host and parasitoid, implying the existence of complex selective pressures on plant investment in chemical defenses, host feeding behavior, and parasitoid life history.

## Introduction

Plant chemical defenses are ubiquitous, and plant-insect coevolution is thought to be a major driver of species diversity [1–2]. However, in many instances plant defenses adversely affect insect herbivore palatability to predators, parasitoid fitness, or even hyperparasitoid fitness [3–7]. These trophic effects can reduce the selective advantage of a costly plant investment in the production of chemical defenses and can change selective pressures on patterns of plant induction and host behavior [8–9]. As a result, an understanding of the trophic consequences of plant chemical defenses is critical to understanding how and when these defenses drive patterns of diversity and structure ecological communities.

Developmental stage has often been observed to play a role in both host-parasitoid interactions and the vulnerability of insect herbivores and their parasitoids to plant defensive compounds [10–14, 6]. Variation across development in the effects of plant chemistry on hosts and parasitoids can lead to different ecological effects of the same plant chemical defense response depending on developmental context [6, 10]. Varying selective pressures on patterns of plant chemical induction, host behavior, and parasitoid life history across host and parasitoid development can favor seasonal or temporal variation in plant chemical defenses [10], variable self-medicating behavior in hosts across development [6], or changes in the timing of parasitoid feeding and development [11–15]. For these reasons, developmental context may play an important role in shaping the evolutionary ecology of plant chemical defenses.

In this study we test the effects of a set of phenolic compounds (flavonoids and hydroxycinnamic acid derivatives) produced by the sensitive fern *Onoclea sensibilis* (Dryopteridaceae) on the shelter-building fern moth *Herpetogramma theseusalis* (Walker) (Lepidoptera: Crambidae) and its abundant braconid parasitoid *Alabagrus texanus* (Cresson) (Hymenoptera: Braconidae). Phenolic compounds constitute a major class of plant secondary metabolites that frequently make plant tissues toxic or indigestible to insect herbivores and are implicated in both constitutive and induced defenses to herbivory [16–19]. This system differs from many other tri-trophic systems in which plant chemical effects have been characterized in that instead of ovipositing into late instar host larvae or pupae, the parasitoid oviposits in early instar larvae immediately after they hatch in the late summer, prior to diapause. Parasitoid larvae remain dormant within the host for nearly their entire development before rapidly destructively feeding upon the host larva just prior to its pupation, a life history strategy referred to as koinobiont development [15]. In addition, the host larvae do not move frequently between plants of different species, but instead feed upon the pinnae contained within leaf shelters of their own construction [20]. The role of trophic effects in such systems is poorly understood [12], although the modes of parasitoid development and host feeding represented in this study are widespread [21, 15]. In this study, we identify a set of phenolic compounds induced by the sensitive fern that affect the growth, development, and survival of the fern moth and its most abundant parasitoid, and determine how the concentration of these compounds varies seasonally and with respect to host shelter-building behavior, in order to determine how selection will act on plant investment and host behavior in this widespread but poorly characterized class of tri-trophic systems.

## Materials and Methods

### Study site

We conducted this study in the summer of 2011 in a 3.5 ha old field at the Darling Marine Center in South Bristol, Lincoln County, Maine, USA (43°56’ N, 69°33’ W), with the permission of the University of Maine. This study did not include any endangered or protected species. Ferns grow densely in the wetter parts of the field. Sensitive fern is the most common fern species in the field, followed by marsh fern *Thelypteris palustris* (Thelypteridaceae); a few other fern species occur in extremely low numbers [20]. We collected all fern moth larvae from two 16 square meter plots of dense sensitive fern approximately 30 m apart, and we collected all fern material immediately adjacent to these plots in areas containing only sensitive ferns. Since adult moths and wasps move freely between and beyond these two plots we have therefore treated them as members of a single community.

### Organisms

Sterile fronds of the sensitive fern average 70 cm tall by a maximum of 30 cm wide and present a roughly triangular shape, characterized by wings of leafy tissue joining the pinnae. The densest patches of sensitive fern in our study site grow in seasonally wet, unshaded areas of the field. They emerge during the first few days of June at the study site [20].

Adult fern moths fly from mid-July to late August and oviposit in the leaf shelters that their generation constructed, as well as on the lower pinnae of the ferns. Larvae hatch in August and feed on these ferns until the onset of dormancy in late summer, overwinter in the litter, and emerge with the young ferns in late spring. They then move to the apices of the ferns, first folding over the tip and subsequently forming a roughly spherical shelter from the ferns’ distal pinnae. The larvae live in and feed on these shelters, occasionally abandoning a shelter and constructing a new one (as evidenced by the continued appearance of new shelters containing large larvae throughout the summer), until they reach a mass of approximately 100–140 mg and pupate within the shelter.

The fern moth’s most common parasitoid at our study site is the braconid koinobiont endoparasitoid *Alabagrus texanus*, which oviposits into young fern moth larvae in late summer and emerges as a larva just prior to fern moth pupation during the following summer. After emergence the parasitoid larva quickly spins a cocoon and pupates, eclosing on average a few days after the fern moths [20]. *Alabagrus* regularly parasitize 50–70% of their hosts [20]. We have found no evidence that the fern moths encapsulate these parasitoids.

### HPLC analysis of phenolic compounds

We collected five samples from each fern type every 10 days starting on 10 June (the last samples, on 18 August, were collected only nine days after the previous collection because of time constraints). In all but the last two collections, we took shelter and shelter-adjacent samples only from shelters that contained feeding larvae. In the last two collections, nearly all individuals had pupated, so we took samples from shelters that were not being actively fed upon. We immediately froze the samples at -80°C and then transported them on dry ice. We extracted phenolic compounds from the samples with 80% aqueous methanol and analyzed extracts by reversed phase HPLC on a 1100 series instrument (Agilent, Foster City, CA) equipped with a diode array detector and a C18 column (150 x 4.6mm, 3um, Gemini, Phenomenex, Torrance, CA) as described by Keinanen et al. [22]. We classified isolated peaks into compound classes based on their UV absorption characteristics and quantified hydroxycinnamic acid derivatives and flavonoids by integration of UV absorption signals at 320 and 360nm, respectively.

### Collections and treatments

We monitored the study area daily for larval emergence, starting on 1 June; the first individuals emerged shortly after. We collected all emerging larvae from the shelters they constructed on the day they emerged. We did not include larvae that initially weighed over 30 mg, because we could not account for food quality over their early development.

We separated all larvae collected each day into individual vials exposed to natural lighting in the laboratory containing approximately the amount of fern material found in a shelter, with the type of fern material provided corresponding to seven feeding treatments, as follows. In total, each treatment was applied to approximately 80 individuals. Individuals were fed every other day, at which time we removed all previously uneaten food, regardless of how much had been consumed, to track seasonal changes in fern composition. We fed three collections the same type of sensitive fern material during the complete developmental process: one received distal pinnae (analogous in position to those used in shelter construction) from ferns with no evidence of herbivory; the second, pinnae from within shelters; and the third, pinnae adjacent to shelters. In the other four treatments, we varied food type across host development. We split host larval development into three segments: early development up until the first day that larval mass exceeded 35 mg, mid development—until the first day that larval mass exceeded 50 mg, and late development—until death or pupation. We chose these masses because an earlier pilot experiment provided evidence that larvae collected from the field in the 35–50 mg range varied in their parasitism rate depending on whether they had been fed shelter material or pinnae with no evidence of herbivory. We switched the food between no-herbivory and shelter-adjacent pinnae as opposed to between no-herbivory and within-shelter pinnae in these treatments to maximize the probability of detecting the effects of induced plant defenses in case these defenses were partially inactivated by shading or some other aspect of shelter construction [23]. When larvae approached maximum size, we monitored them each day to determine their exact date of pupation and eclosion (median larval duration = 19 days, median pupal duration = 12 days). We frequently observed large dead parasitoid larvae that failed to emerge from dead host larvae.

### Dissections

We collected healthy-looking fern moth larvae haphazardly in areas adjacent to our established sites from 26 June to 28 June, shortly before pupation of the earliest-maturing larvae, and dissected them in order to explore the relationship between host and parasitoid growth. The larvae ranged in size from 10.7 mg to 121.7 mg and thus represented all but the very smallest and the very largest larvae found at any point throughout the season.

### Data analysis

We used hierarchical clustering of Pearson correlation distances between the HPLC readings across fern tissue samples for the 11 phenolic compounds analyzed to identify a cluster of 7 compounds that covaried strongly. We performed principal components analysis on the scaled intensities of these 7 compounds, and used their first principal component as an index of the overall state of phenolic induction for a given fern tissue type and time point with respect to this dominant source of chemical variation. We used two-way analysis of variance (ANOVA) to test the effects of differences between fern tissue types and season on phenolic induction.

Because fern phenolic content varied widely across time and tissue type over the course of the experiment, we sought to test whether differences in exposure to these compounds across development better explained variation in outcomes than a model based on just fern tissue types. We estimated the exposure of individual larvae during each developmental stage (<35 mg, 35–50 mg, >50 mg) to PC1 of the main phenolic cluster of compounds by interpolating PC1 values between sampling dates for each fern tissue type and taking the mean of these values across the days for which each larva was in a particular developmental stage. Using the R package nnet, we fit multinomial logistic models of outcome for larvae that survived to a mass >50 mg (host pupa, parasitoid pupa, or dead larva) as a function of food during early, middle, and late development, accounting for the effects of different starting larval masses as well as collection date. We used Akaike Information Criterion (AIC) values to compare the phenolic exposure model to the model based on just fern tissue types. In addition, we used a binomial generalized linear model (GLM) to test whether exposure to PC1 of the main phenolic cluster increased the incidence of early larval mortality (<35 mg). We used backwards stepwise variable selection to fit linear models, accounting for the effects of collection date and starting larval mass, to test the effects of phenolic exposure during early, middle, and late development on pupal mass, adult mass, time to pupation, and time to eclosion for both host and parasitoid. We carried out all analyses in R [24].

## Results

**Fig 1:**
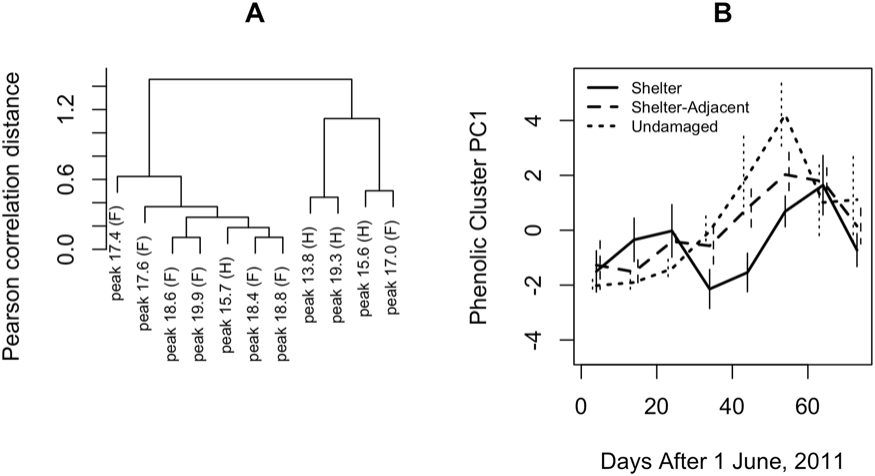
Chemical relationships between fern tissues vary seasonally. (A) Seven of the 11 phenolic compounds analyzed covaried in concentration across fern samples. Compounds are classified as flavonoids (F) or hydroxycinnamic acid derivatives (H). (B) The concentrations of these compounds in undamaged, shelter-derived, and shelter-adjacent fern tissues changed across the season, and the relative quantities in shelter-derived and undamaged fern tissues switched in the mid-summer.

We found that seven of the 11 phenolic compounds, 6 flavonoids and 1 hydroxycinnamic acid derivative, showed highly correlated concentrations across all analyzed fern tissue samples (Fig. 1A). We summarized the overall level of these compounds in a given sample as the first principal component in a principal components analysis of their scaled intensities across samples, which accounted for 75% of variance in these compounds. Among samples collected while fern moth larvae were still feeding, these compounds varied according to a strong interaction between fern tissue type and season (Fig. 1B, two-way ANOVA P<0.001), with undamaged fern material having lower levels at the beginning of the summer and higher levels at the end. In the late summer, when larvae were no longer feeding, phenolic levels dropped in all fern tissue types.

**Fig 2:**
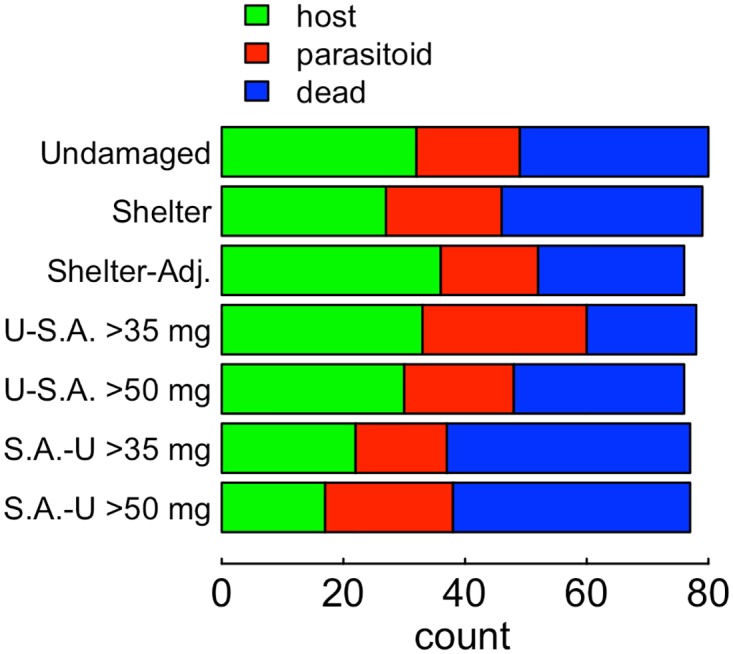
Developmental outcomes of different feeding treatments. Undamaged food was made up of distal pinnae (analogous in position to those used in shelter construction) from ferns with no evidence of herbivory. Shelter food was made up of pinnae from within shelters, and shelter-adjacent, pinnae adjacent to shelters. Switched treatments were switched between undamaged (U) and shelter-adjacent (S.A.) food in the direction and on the first day they exceeded either 35 or 50 mg, as indicated.

The different feeding treatments yielded different proportion of host pupae, parasitoid pupae, and dead larvae (*X*-squared = 26.8, df = 12, *P*<0.01, Fig. 2). Because we switched larvae between fern tissue types at different points in development, and the chemistry of these tissues varied widely across the experiment, larvae experienced a wide range of chemical trajectories across the experiment, allowing us to isolate the effects of plant chemistry on both host and parasitoid at different points in fern moth development. A multinomial logistic model of developmental outcome (moth, parasitoid, pupa) based on exposure during each developmental stage to the main cluster of phenolic compounds explained the data substantially better than a model based on just fern tissue type consumed at each developmental stage (ΔAIC = 7.5, Table 1). Exposure to the correlated set of phenolic compounds in early development was associated with increased mortality of larvae under 35 mg (Table 2, binomial GLM *Z* = 2.7, P<0.01). Among larvae that survived to 50 mg, exposure to this set of phenolic compounds in early development (<35 mg) was associated with decreased parasitoid survival (Table 1, multinomial logistic model *Z* = -2.8, *P*<0.01). Larvae exposed to this set of phenolic compounds between 35 and 50 mg yielded more parasitoid larvae (Table 1, multinomial logistic model *Z* = 3.7, P<0.001). Larvae exposed to this set of phenolic compounds during during late development (>50 mg) yielded fewer parasitoid larvae (Table 1, multinomial logistic model *Z* = -2.6, *P*<0.05).

**Table 1:** Multinomial logistic model of the effects of a cluster of phenolic compounds on host and parasitoid survival across development among larvae that survived to at least 50 mg.

	Host Coef.	Host S.E.	Parasitoid Coef.	Parasitoid S.E.
**Intercept**	0.69	0.61	-0.49	0.66
**Collection date**	-0.12	0.02	0.00	0.02
**Mass at collection**	0.07	0.02	0.00	0.02
**Phenolic exposure <35 mg**.	-0.69	0.40	-1.15	0.41
**Phenolic exposure 35–50 mg**.	0.47	0.46	1.82	0.48
**Phenolic exposure >50 mg**.	0.36	0.37	-1.02	0.40

**Table 2:** Binomial generalized linear model of the effects of early developmental mortality (<35 mg) as a function of exposure to a cluster of phenolic compounds.

	Coefficient	Standard Error
**Intercept**	0.55	0.47
**Collection date**	-0.02	0.02
**Mass at collection**	-0.07	0.01
**Phenolic exposure < 35 mg**.	0.69	0.25

We also tested the effects of fern tissue phenolic content on the duration of larval development and the mass of host pupae, as well as the mass of both host and parasitoid adults. Exposure to the cluster of phenolic compounds during late development was associated with smaller host pupae (-8.1 mg/unit PC1 phenolic cluster, unit PC1 as shown in Fig. 1, *t* = -3.6, df = 186, *P*<0.001). This effect was diminished among surviving adults (-0.9 mg/unit PC1 phenolic cluster, *t* = -1.2, df = 143, *P* = 0.2), due to increased pupal mortality among smaller pupae (binomial GLM effect = -0.03/mg, *Z* = -2.1, *P*<0.05). Exposure to the cluster of phenolic compounds was also associated with later pupation (1.34 days/unit PC1 phenolic cluster, *t* = 3.6, df = 192, *P*<0.001) and eclosion (1.48 days/unit PC1 phenolic cluster, *t* = 3.3, df = 154, *P*<0.01) among fern moths and parasitoids (pupation: 1.13 days/unit PC1 phenolic cluster, *t* = 1.9, df = 100, *P* = 0.05, eclosion: 1.69 days/unit PC1 phenolic cluster, *t* = 3.1, df = 89, *P*<0.01).

**Fig 3:**
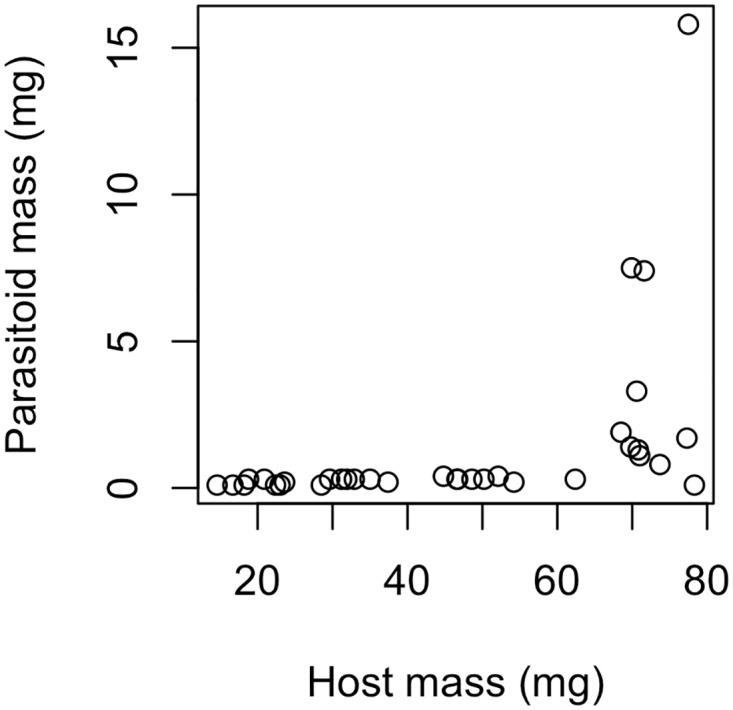
Parasitoid development across the course of host development. Parasitoid larvae remained at a low mass (<1 mg) for most of host development before initiating rapid, destructive feeding and growth in approximately 60–70 mg host larvae.

Among field-collected host larvae dissected across a range of masses, parasitoid larvae remained at a consistently low mass (<1 mg) until hosts reached a mass of 60–70 mg, after which parasitoid larvae initiated rapid, destructive feeding and growth and varied widely in mass between hosts (Fig. 3).

## Discussion

We find that leaf shelters constructed by the fern moth *Herpetogramma theseusalis* feeding on the sensitive fern *Onoclea sensibilis* contain different concentrations of a cluster of co-induced phenolic compounds from undamaged fern material, and that the relative amounts of these compounds in shelters and undamaged fern tissue switch across the summer. These phenolic compounds had variable effects on the fern moth and its abundant parasitoid *Alabagrus texanus* across host and parasitoid development (Table 1). During early fern moth larval development, exposure to these compounds was associated with increased mortality, reducing both moth and parasitoid survival overall, as well as lower parasitism rates among surviving larvae, consistent with previous studies showing vulnerability of early instar larvae to plant defensive compounds [10].

As the moth larvae grew larger, exposure to these compounds was not associated with increased larval mortality, but was associated with increased parasitoid survival (Table 1). In the final stages of larval development, when parasitoids exit apparent developmental stasis and begin explosive growth, exposure to these phenolic compounds was associated with lower parasitoid survival (Table 1, Fig. 3). These results show that not only can plant defensive compounds affect the fitness of parasitoids feeding on insect herbivore hosts, but that the direction of these effects can vary across development. This observation is consistent with previous work demonstrating variation in host self-medicating behavior across development [6]. These phenolic compounds likely remained somewhat toxic to both host and parasitoid across development, since both hosts and parasitoids exposed to higher amounts of the compounds took longer to develop and host pupae were smaller and experienced higher levels of pupal mortality, consistent with other studies of the effects of induced plant compounds on host and parasitoid development [25–26]. We do not know the physiological mechanism of the mid-developmental switch to positive effects of phenolic exposure on parasitoid survival, but it may be related to the effects of plant chemical defenses on the ability of hosts to prevent parasitoid development [6].

Host feeding ecology is an important selective force on the evolution of parasitoid developmental strategies [12,13], and variation in host quality across development has frequently been observed to shape parasitoid life history and ecology [11–15]. Our results suggest that variation in plant chemistry related to both seasonal and host behavioral effects may change the relationship between host quality and host developmental stage across space and time. Such variation could exert strong selection on the rate and timing of parasitoid development [12, 15]. These findings agree with previous work suggesting that trophic effects may play an important role in parasitoid life history evolution [12].

The relative roles of fern moth shelter-building behavior, patterns of fern induction, or even parasitoid manipulation of host behavior in determining the chemical makeup of leaf shelters remain an intriguing open question [27–29, 17]. The higher levels of the main cluster of phenolic compounds in early season shelter and shelter-adjacent fern material suggests that shelter-building behavior does not allow the fern moth to escape the negative effects of plant chemical defenses in its early development. The abrupt mid-summer drop in the level of the identified phenolic compounds in shelter-derived fern material should have strong effects on parasitoid survival, although we cannot determine the most common effects in the field without tracking the chemical trajectories of individual shelters across host development. A reduction earlier in host development should decrease parasitoid survival, whereas the same reduction later in host development should increase parasitoid survival (Table 1). Future studies should determine the relative roles of host behavior and plant induction patterns in generating this seasonal variation in leaf shelter chemistry.

In conclusion, we find that the chemical makeup of the sensitive fern varies widely according to an interaction between seasonal effects and the effects of shelter construction and feeding by the larvae of the fern moth. This chemical variation had variable effects across development, including both positive and negative effects on the survival of parasitoid larvae at different points in host development. These results highlight the complexity of the leaf shelter chemical environment, and demonstrate the importance of developmental context for determining the effects of plant defenses on multiple trophic levels.

## Supporting Information

S1 File Raw HPLC data, normalized to fern fresh weight. (CSV)

S2 File Raw data from experimental feeding treatments. (CSV)

S3 File Raw data from dissections of field collected larvae. (CSV)
